# *ERCC1*, *XRCC1*, and *GSTP1* Polymorphisms and Treatment Outcomes of Advanced Epithelial Ovarian Cancer Patients Treated with Platinum-based Chemotherapy

**DOI:** 10.31557/APJCP.2020.21.7.1925

**Published:** 2020-07

**Authors:** Salisa Liblab, Apichai Vasuratana, Nutthada Areepium

**Affiliations:** 1 *Department of Pharmacy Practice, Faculty of Pharmaceutical Sciences, Chulalongkorn University, Bangkok, Thailand. *; 2 *Department of Obstetrics and Gynecology, Faculty of Medicine, Chulalongkorn University, Bangkok, Thailand. *

**Keywords:** Ovarian cancer, platinum chemotherapy, polymorphisms

## Abstract

**Objective:**

The first line regimen for treating epithelial ovarian cancer (EOC) is platinum-based chemotherapy. Various factors impact its effectiveness including polymorphisms of enzymes in platinum-related metabolism processes.

**Methods:**

We conducted the study to investigate the association between polymorphisms of *ERCC1*, *XRCC1* and *GSTP1*, which responsible for platinum’s metabolisms in Thai epithelial ovarian cancer patients.

**Results:**

Fifty-two patients with advanced epithelial ovarian cancer were enrolled into this study. Genotyping analysis of *ERCC1* (C->A, *rs3212986*), *XRCC1 *(*A->G, rs25487*) and *GSTP *(*A->G, rs1695*) were performed which variant allele frequencies were found at 35.6%, 28.9% and 10.6%, respectively. Patients with homozygous variant type (A/A) of *ERCC1 C8092A* had higher rate of platinum-resistance (75% vs 16.7%, p =0.046). In addition, the significant association of GSTP1 polymorphism and grade 2 anemia was found. Patients with A/G genotype of GSTP1 had higher rate of grade 2 anemia (81.8% vs 46.3%, p =0.036).

**Conclusions:**

Genetic polymorphisms of *ERCC1*, and *GSTP1* might be useful biomarkers for prediction of clinical benefit and toxicities of platinum-based chemotherapy in Thai epithelial ovarian cancer patients.

## Introduction

Epithelial ovarian cancer is the major histology of ovarian malignancies that exist around 90%. Ovarian cancer was the 7^th^ most common cancer and the 8th most common causes of cancer death in women which reported in world cancer statistics. Because of the location, it is difficult to diagnose, most patients have been diagnosed at advanced stage with 5-year survival rate lower than 45% (Webb and Jordan, 2017). The National Health and Security Office, Thailand suggested that epithelial ovarian cancer should be treated by platinum-based chemotherapy, which is cisplatin or carboplatin, similar to the United States’National Comprehensive Cancer Network (NCCN).

Platinum compounds are alkylating agents that damage cancer cell by inhibition of DNA inter-strand and intra-strand crosslink including to DNA protein crosslink (Khrunin et al., 2010). These mechanisms are associated with many genes such as excision repair 1 (*ERCC1*), a gene in Nucleotide excision repair (NER) system, which remove DNA lesion from platinum compounds or X-ray repair complementing 1 (*XRCC1*), a gene in Base excision repair (BER) system, which remove base in DNA single strand lesion. Both genes are excise and repair platinum compound adduct at inter- and intra-strand of DNA. Moreover, detoxification pathway in cell cytoplasm associated with the glutathione S-transferase protein which encoded by glutathione S-transferase pi 1 (*GSTP1*). The conjugation between platinum compounds and *GSTP1 *resulted in depleted cytotoxic activity. Previous studies reported that the polymorphisms of these genes had been associated with clinical response and adverse events from platinum-based chemotherapy (Marsh, 2009; Galluzzi et al., 2012).

The association between genetic polymorphisms of *ERCC1*, *XRCC1* and *GSTP1* with progression free survival (PFS) and overall survival (OS) in patients with epithelial ovarian cancer in varies ethnicities such as American, European or Asian were reported in several studies (Kim et al., 2009; Khrunin et al., 2010; Steffensen et al., 2011; Kang et al., 2013; Li and Li, 2013). However, the results from prior studies had been inconsistent. Therefore, research to investigate the association of *ERCC1*,* XRCC1* and *GSTP1* polymorphisms with clinical treatment responses and adverse events from platinum-based chemotherapy especially in Thai patients with advanced epithelial ovarian cancer was needed.

## Materials and Methods

Fifty-two advanced epithelial ovarian cancer patients received platinum-based chemotherapy at King Chulalongkorn Memorial hospital during May to September 2018 were enrolled. Eligible patients had a histopathology confirmation for stage III or IV epithelial ovarian cancer classified by the International Federation of Gynecology and Obstetrics (FIGO) system and treated with intravenous platinum-based chemotherapy. Patients received 6 cycles of chemotherapy consisting of single carboplatin, which selected Area Under the Curve (AUC) as 6 every 4 weeks or carboplatin-based chemotherapy (AUC 5 or 6) and paclitaxel (175 mg/m^2^) every 3 weeks. Five milliliter of blood samples were drawn for genotyping. Clinical benefit was evaluated after completion of chemotherapy per Response Evaluation Criteria in Solid Tumors (RECIST) criteria or at least as documented by physician in patients’ medical record. Adverse events had been assessed according to Common Terminology Criteria for Adverse Events (CTCAE) version 4.03 at every cycle of chemotherapy. All participants gave an informed consent with expressed in the Declaration of Helsinki.Patients were interviewed and medical chart were reviewed to collect relevant clinical information. This study was approved by Institutional Review Board of Faculty of Medicine, Chulalongkorn University, Bangkok, Thailand. The IRB approval number was 162/61.

Genomic DNA was extracted from peripheral venous blood following QIAamp Blood Mini Kit (Qiagen Gmbh, Hilden, Germany) manufacturer’s protocol. The genetic polymorphisms of of *ERCC1* (*C-A, rs3212986*), *XRCC1 *(*A-G, rs25487*) and* GSTP* (*A-G, rs1695*) were performed were determined by the Taqman allelic discrimination assay through polymerase chain reaction (PRC). After polymerase chain reaction amplification, the endpoint plate was read by StepOnePLus Real time PRC system (Applied Biosystems Inc., Foster City, CA USA). The final products were analyzed by using fluorescent signals with Sequence Detection System (SDS) software. 

Statistical analysis was performed using Statistical Package for Social sciences (SPSS) version 22.0 (SPSS. Co., Ltd., Bangkok, Thailand). Patients’ characteristics were reported as mean standard deviation (SD) and percentage (%). The association between polymorphisms and treatment outcomes were analyzed by Chi-squared test or Fisher’s exact test. The p-value < 0.05 was measured two-sides statistically significant.

## Results

A total of 52 patients with Epithelial ovarian cancer, the average age was 55.71+10.52 with ranging from 35 to 85 years old. Most of patients (57.69%) had disease in FIGO stage III. Poorly differentiated serous adenocarcinoma histology was the most common found in 19 patients (36.54%). Main chemotherapy regimen which used in this study was carboplatin (AUC 5 mg × ml/min) combined with paclitaxel (175 mg/m^2^). In this study, there were 27 patients with recurrent disease and previously treated with platinum based chemotherapy. The patients’ characteristics was shown in Table 1.

*ERCC1* polymorphism, (*C>A, rs3212986*) was found as homozygous wild type (C/C), heterozygous variant (C/A) and homozygous variant (A/A) at 48.08%, 32.69% and 19.23%, respectively. *XRCC1 *polymorphism, (*G>A, rs25487*) was found as homozygous wild type (G/G), heterozygous variant (G/A) and homozygous variant (A/A) at 53.85%, 36.54% and 9.61%, respectively. Since *GSTP1* polymorphism (*A>G, rs1695*) was uncommon in this population, therefore only 2 genotypes were found as homozygous wild type (A/A) and heterozygous variant (A/G) at 78.85% and 21.15%, respectively, the data was shown in Table 2. 

Treatment outcome was evaluated after completion of chemotherapy. From 52 patients which were included into this study, 48 patients completed 6 cycles of platinum-based chemotherapy and were evaluated for the responses. Three patients (6.25%) had poor treatment outcome as worsening symptoms or disease progression resulted in changing to other chemotherapy regimen. While most of the patients (45 or 93.75% had better treatment outcomes from the chemotherapy as disease can be controlled or none of worsening symptoms those lead to stop platinum-based chemotherapy). However, all three genetic polymorphisms were not associated with treatment outcome while only tumor debulking status was associated with favorable outcome (p=0.006) as revealed in Table 3. 

In our study, there were 27 cases with disease recurrences, there were 7 patients (25.92%) identified as platinum resistance which the cancer recurrences within 6 months after completion of platinum-based chemotherapy treatment.

Interestingly, polymorphism of *ERCC1* seemed to have impact on platinum resistance. Patients with variant alleles had higher risk of platinum resistance. Patients with A/A genotypes had greater rate of resistance when compared with patients who had at least one wild type C allele, C/C and C/A, (75.00% vs. 16.67%, p=0.046). However, we did not found any associations of *XRCC1 *and *GSTP1* polymorphisms and disease resistance rate as shown in Table 4.

For the adverse events, the most common hematologic adverse event occurred in this study was anemia (51 cases, 98.08%), followed by neutropenia (37 cases, 71.15%) and thrombocytopenia (20 cases, 38.46%). We found that patients with variant genotype (A/G) of *GSTP1* had higher risk of grade 2 anemia than wild type (A/A) which presented in 81.82% compared with 46.34% (OR=5.2; 95%CI: 1.000-27.146; =0.036), as presented in Table 5. No other significant association were found between genetic polymorphisms and hematologic adverse events.

**Table 1:** Demographic and Clinical Characteristics of Patients

Characteristic	N (%)	
Characteristics	n	%
FIGO stage		
Stage III	30	57.69
Stage IV	12	23.08
N/A	10	19.23
Performance status		
ECOG score=0	30	57.69
ECOG score=1	19	36.54
ECOG score=2	3	5.77
Histological type		
Serous adenocarcinoma	28	53.85
Endometrioid adenocarcinoma	8	15.38
Clear cell carcinoma	5	9.62
Mucinous adenocarcinoma	1	1.92
Mixed	4	7.69
N/A	6	11.54
Tumor grading		
Well differentiated	4	7.69
Moderate differentiated	6	11.54
Poorly differentiated	26	50
Unidentified or no data	16	30.77
N/A	10	19.23
Debulking status		
Complete	16	30.77
Optimal	21	40.38
Suboptimal	9	17.31
No surgery	4	7.69
N/A	2	3.85
Chemotherapy regimen		
Single carboplatin	3	5.77
Carboplatin + Paclitaxel	49	94.23

**Table 2:** Frequency of *ERCC1, XRCC1* and *GSTP1* in Thai EOC Patients

Total number of participants = 52	Number	Percent
*ERCC1 C8092A *genotype		
Homozygous wild genotype (C/C)	25	48.08
Heterozygous variant genotype (C/A)	17	32.69
Homozygous variant genotype (A/A)	10	19.23
*XRCC1 A399G* genotype		
Homozygous wild genotype (G/G)	28	53.85
Heterozygous variant genotype (G/A)	19	36.54
Homozygous variant genotype (A/A)	5	9.61
*GSTP1 Ile105Val* genotype		
Homozygous wild genotype (A/A)	41	78.85
Heterozygous variant genotype (A/G)	11	21.15
Homozygous variant genotype (G/G)	0	0

**Table 3:** Association of Genetic Polymorphisms and Clinical Outcomes

Gene	N	Treatment outcome		*P*-value
		Good	Poor	
		(N=45, %)	(N=3, %)	
*ERCC1*				0.132
CC	23	22 (95.65)	1 (4.35)	
CA	15	15 (100.00)	0 (0.00)	
AA	10	8 (80.00)	2 (20.00)	
*XRCC1*				0.204
GG	25	23 (92.00)	2 (8.00)	
GA	18	18 (100.00)	0 (0.00)	
AA	5	4 (80.00)	1 (20.00)	
*GSTP1*				0.512
AA	38	36 (94.74)	2 (5.26)	
AG	10	9 (90.00)	1 (10.00)	

**Table 4:** Association of Genetic Polymorphisms to Resistance Status

Gene	N	Resistance		*P*-value
		Non-Resistance (N=20, %)	Resistance (N=7, %)	
*ERCC1*				0.046*
CC+CA	18	15 (83.33)	3 (16.67)	
AA	4	1 (25.00)	3 (75.00)	
*XRCC1*				1
GG+AA	18	13 (72.22)	5 (27.78)	
AA	4	3 (75.00)	1 (25.00)	
*GSTP1*				1
AA	20	15 (75.00)	5 (25.00)	
AG	7	5 (71.43)	2 (28.57)	

**Table 5:** Association of Genetic Polymorphisms and Anemia

Gene	N	Anemia (grade)		*P*-value
		Grade 0,1 (N=24, %)	Grade 2 (N=28, %)	
*ERCC1*				0.61
CC	25	11 (44.00)	14 (56.00)	
CA	17	7 (41.18)	10 (58.82)	
AA	10	6 (60.00)	4 (40.00)	
*XRCC1*				0.356
GG	28	12 (42.86)	16 (57.14)	
GA	19	8 (42.10)	11 (57.90)	
AA	5	4 (80.00)	1 (20.00)	
*GSTP1*				0.036*
AA	41	22 (53.66)	19 (46.34)	
AG	11	2 (18.18)	9 (81.82)	

## Discussions

This is the first study that explore both prevalence and impact of polymorphisms of platinum drug related metabolizing enzymes in Thai epithelial ovarian cancer patients. We found rather higher prevalence of *ERCC1* *C8092A* at 35.6% compared to which reported in Korean population reported as 25.9% (Kim et al., 2009). While this polymorphism found around 24-29% in Caucasians and Europeans (Krivak, 2011; Steffensen, 2011). For *XRCC1 A399G*, the previous report in Thai cervical cancer patients was 22% (Ishida et al., 2011) which quite similar to 28.9% in our findings. Which a bit less than In other Asian population. Variant allele frequency in epithelial ovarian cancer patients was found as 24.6% in Korean and 38.7% in Chinese (Kim et al., 2009; Cheng et al., 2012). In our study, we found only 10.6% of variant alleles of *GSTP1 Ile105Val*, contrary to the other report in Thai non-small cell lung cancer patients which variant allele frequency were reported as 26.1% (Kumpiro, 2015). Variant allele frequency in Chinese epithelial ovarian cancer patients was shown rather higher as 29.3% (Zhai et al., 2016). While in Korean epithelial ovarian cancer patients showed quite similar prevalence with our study at 11.4% (Kim et al., 2009). 

As in previous reported, *ERCC1*, *XRCC1* and *GSTP1 *polymorphisms had impacts on treatment responses of platinum based chemotherapy among epithelial ovarian cancer patents. Nonetheless, we found no association among those polymorphisms and treatment outcome. This could be the resulted of limited number of cases. But our interested finding was the association of *ERCC1* polymorphism and platinum resistance among AA carriers than in patients with homozygous wild type and heterozygous variants (75 vs 16.8%, p=0.046). in whom with homozygous variant compared with other genotypes). This was similar to the result from meta-analysis which reported association of progression-free survival (HR = 1.39, 95% CI = 1.12–1.73) and that the CA or AA genotypes could influence overall survival (HR = 1.28, 95% CI = 1.05–1.56; and HR = 1.55, 95% CI = 1.17-2.05, respectively) (Yan et al., 2014). However, this is in direct comparable in terms of clinical outcomes. 

The other positive findings which found in our study was the association between *GSTP1* polymorphism (*A>G, rs1695*) and more severe anemia which consistent with Korean and Japanese studies (Kim et al., 2009; Yoshihama et al., 2018). However, the previous reports indicated that patients who carried homozygous A/A had higher risk of more severe hematologic toxicity which were opposite to our finding. We found those who carried variant genotype (A/G) had significantly higher risk of grade 2 anemia than patients with homozygous wild type (81.82% vs 46.34%, p=0.036). 

In conclusion, findings from this study were explained possible source of variation in the treatment outcomes of platinum in Thai epithelial ovarian cancer patients. Prevalence of *ERCC1*,* XRCC1 *and *GSTP1* were 35.6%, 28.9% and 10.6%, respectively which were not less. Some important associations of* ERCC1*, *XRCC1* and *GSTP1* polymorphisms of clinical outcomes and adverse events in Thai epithelial ovarian cancer patients were noticed. *ERCC1* polymorphism were associated with platinum resistance, while *GSTP1* polymorphism was associated with grade 2 of anemia. However, the small sample size and short period of follow-up was the limitation of this study. Thus, the number of patients in each genotype was limited as a result of under power of statistic testing. Future studies with larger sample size might be presented more impact of these genetic polymorphisms on treatment outcomes and toxicities.
